# A new flowchart for parameters calculation of Hybrid Active Power Filter with Injection Circuit

**DOI:** 10.1371/journal.pone.0253275

**Published:** 2021-07-26

**Authors:** Tung Khac Truong, Chau Minh Thuyen

**Affiliations:** 1 Faculty of Information Technology, Van Lang University, Ho Chi Minh, Vietnam; 2 Faculty of Electrical Engineering Technology, Industrial University of Ho Chi Minh City, Ho Chi Minh, Vietnam; Torrens University Australia, AUSTRALIA

## Abstract

This paper presents a new flowchart for parameters calculation of Hybrid Active Power Filter with Injection Circuit (IHAPF). The first is the necessity to use the IHAPF model and the parameters of the IHAPF needed to search have been shown. Next, the constraints of the parameters to be searched and the objective function to be reached are given. Since then a flowchart is designed to look for parameters of IHAPF using the Jaya optimization algorithm. The Jaya algorithm has the advantage of simplicity, few parameters, and good performance. Therefore it reduces search time. Compared to the flowchart using the firefly algorithm, particle swarm optimization algorithm, and simulated annealing algorithm, the simulation results performed on an IHAPF 10kV-50Hz model have proven that: the proposed flowchart gives better results in minimizing the compensation errors, minimum phase shift angle between supply current, and source voltage, minimum total harmonic distortion of supply current.

## Introduction

As we all know, harmonics that exist on the power system will cause many very serious problems such as: overheating, overload, information interference, etc. Accordingly, to cancel harmonic problems and improve power factors in power systems, the Hybrid Active Power Filter with Injection Circuit (IHAPF) model is considered as one of the most effective solutions [1–5]. IHAPF also has many different structures to use for many different voltage levels, many different types of loads.

The problem here is that once the IHAPF structure is selected, the most important issue is the correct calculation of the parameters of an IHAPF system. The IHAPF is a hybrid of Active Power Filter (APF), and Passive Power Filters (PPF) [6–8]. Therefore, the parameters of an IHAPF usually include the parameters of the passive circuit part, and the parameters of the active circuit part. Passive circuit part parameters are parameters of a serial resonance circuit LR-C at a certain harmonic frequency, often resonating at high order harmonics 11^*th*^, and 13^*th*^. The parameters of the active circuit part include parameters of the injection circuit LR-C, the output filter of the inverter, the DC-link voltage of the inverter, and the parameters of the Proportional–Integral (PI) controller. All IHAPF parameters shall be designed such that it satisfies all the constraints of the parameters, and minimum total harmonic distortion of the supply current, minimum the compensation error, and minimum phase shift angle between the supply current, and source voltage in steady state. This is a multi-objective optimization design problem.

Accordingly, to solve multi-objective optimization problems in general, the Genetic Algorithm (GA) [9, 10], and Particle Swarm Optimization (PSO) [11–13] algorithms are most often used. When applied to the IHAPF system, the GA algorithm is often used to design multi-objective optimizations for PPF parameters [14–17]. Meanwhile, the PSO algorithm is also used for designing PPF parameters but more than the GA algorithm because it usually gives better results [18–23]. However, the PSO algorithm also has the disadvantage that the search space is limited. Therefore, several studies have used a combination of PSO, and GA algorithms [24, 25]. A few studies have also applied Bat [26], and Ant Colony algorithms [27] to design multi-objective optimization of PPF parameters. The latest research, which is highly accurate, and efficient, has been applied to HAPF to find out PPF parameters to minimize the total harmonic distortion of source current, and source voltage [28, 29].

In summary, the multi-objective optimization methods applied to HAPF have largely found an optimal set of parameters PPFs for HAPF. However, the above methods are limited by the slow search speed, and especially only the parameters for the passive circuit part, and the parameters of the active circuit part are not mentioned. Therefore, this paper introduces a new multi-objective optimization design flowchart for the Hybrid Active Power Filter with Injection Circuit using the Jaya algorithm [30–32]. A firefly algorithm(FA) is proposed for Stochastic Test Functions and Design Optimisation in [33]. Jaya has been successful in solving a large range of optimization problems such as Optimal power flow, constrained Multi-objective optimization problem, optimize parameter for a neural network [34–37]. The advantage of this algorithm is simple, giving fast results. To prove the effectiveness of the proposed design flowchart, the simulation results were performed on the same model with the application of the Jaya, firefly algorithm, particle swarm optimization algorithm, and simulated annealing(SA) algorithm. The simulation results have proved that: the flowchart of applying Jaya algorithm give parameters set is better than that of applying firefly algorithm, particle swarm optimization algorithm, and simulated annealing algorithm in minimizing the total harmonic distortion of supply current, minimizing compensation error, minimize the phase shift angle between supply current, and source voltage, and especially, the calculation time is shorter.

The paper is structured in four parts: Part 1 presents the necessary of the problem to be studied, part 2 introduces the model, and the parameters needed to design, part 3 introduces the multi-objective optimization design flowchart for IHAPF using the Jaya algorithm, the simulation, and discussion results are presented in part 4, and finally the conclusions are drawn in part 5.

## IHAPF model, and necessary design parameters

**Fig 1:**
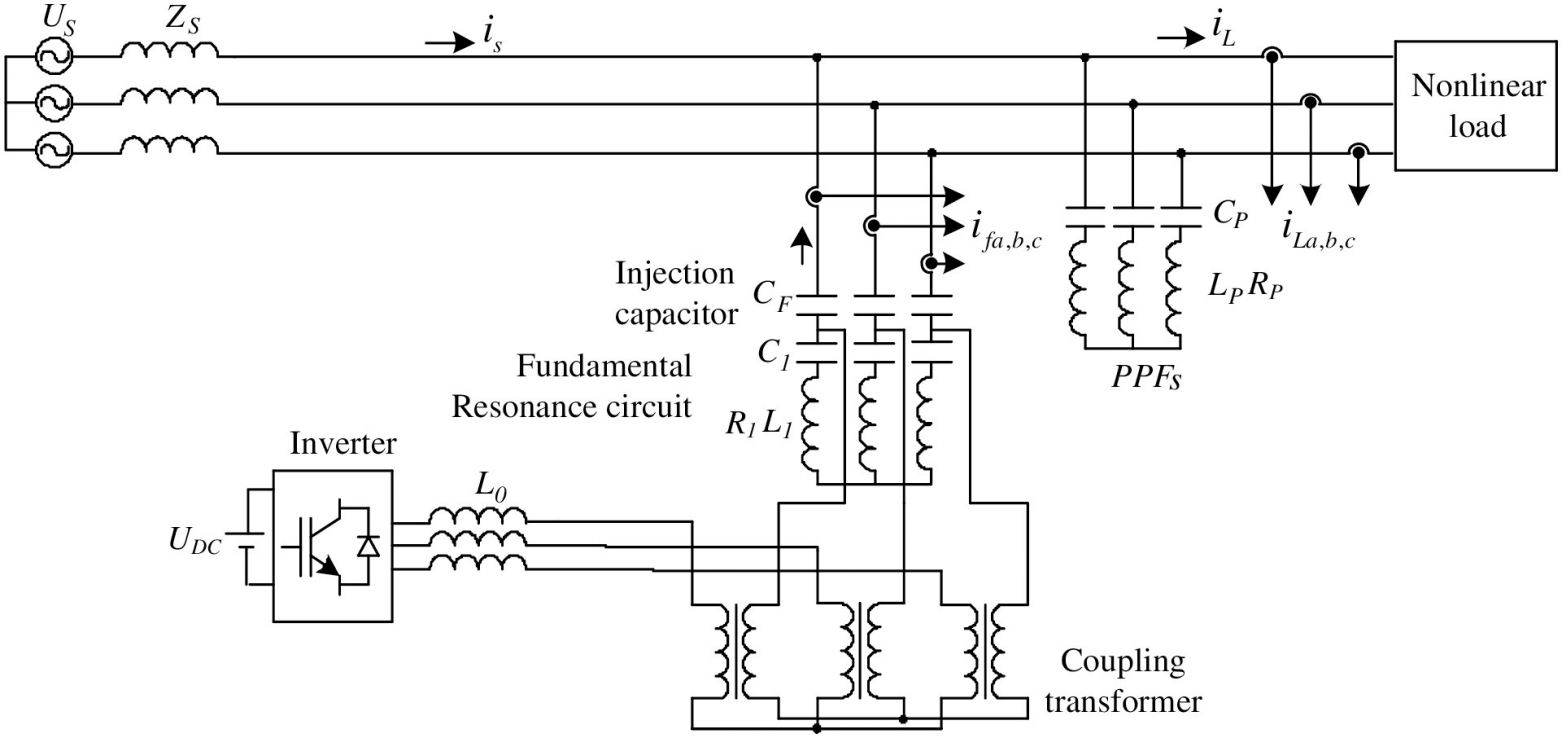
Structure of an IHAPF.

Let us consider a model IHAPF shown in Fig 1.

The parameters of the passive circuit part include: *C*_*P*_, and *L*_*P*_ *R*_*P*_ are the capacitors, and inductors of passive power filters (*R*_*P*_ is the internal resistance of *L*_*P*_), it used to filter high order harmonics. *C*_*F*_ is the injection capacitor, the *L*_1_, and *C*_1_ are the fundamental resonance capacitor, and inductor, the *L*_1_*R*_1_ − *C*_1_ branch resonates at the fundamental frequency, (*R*_1_ is the internal resistance of *L*_1_). *C*_*F*_ combined with *L*_1_*R*_1_ − *C*_1_ forms an injection circuit that to filter harmonics, compensate reactive power. Furthermore, it reduces the voltage applied to the inverter. Therefore the capacity of the inverter is reduced. The coupling transformer is used to isolate the system, and the inverter. The output filter *L_0_* has the effect of reducing the voltage spikes at the output of the inverter when the inverter is switching at high frequency. The inverter used in this model is a voltage source inverter with a *U*_*DC*_ power supply. Parameters of the active circuit part include: *K*_*p*_, and *K*_*i*_ of the PI controller.

**Fig 2:**
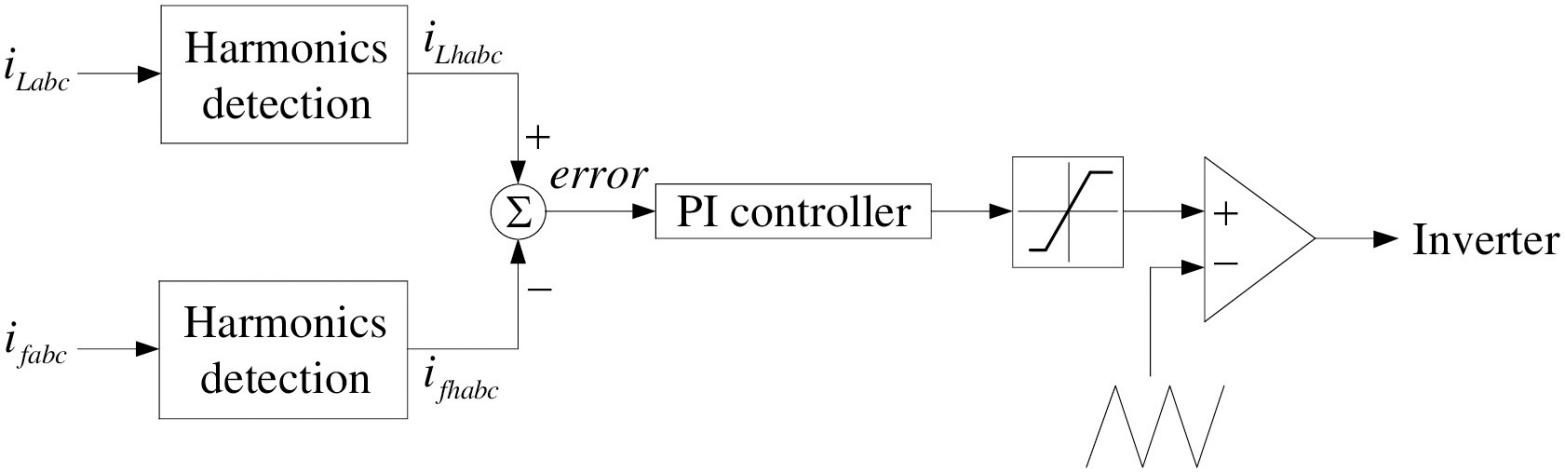
IHAPF control block diagram.

Control block diagram for IHAPF is described in Fig 2. The three-phase load current *i*_*Labc*_ through the *i*_*p*_ − *i*_*q*_ harmonic detection circuit [20] to separate harmonic components *i*_*Lhabc*_. These are reference signals. The compensation current *i*_*fabc*_ is also connected to the *i*_*p*_ − *i*_*q*_ harmonic detection circuit to separate harmonics components *i*_*fhabc*_, which are considered as actual signals. The error between the actual signals, and the reference signals will be passed through the PI controllers (including the parameters *K*_*p*_, and *K*_*i*_) to minimize this error. The output of the PI controller will be compared with the high frequency carrier wave to create pulse into inverter. Thus, the parameters to be determined for the IHAPF system include: *C*_F_ (F), *R*_1_(Ω), *L*_1_ (H), *C*_1_ (F), *R*_11_ (Ω), *L*_11_ (H), *C*_11_ (F), *R*_13_ (Ω), *L*_13_ (H), *C*_13_ (F), *L*_0_ (H), *U_DC_* (V), *K_p_*, and *K*_*i*_.

## Multi-objective optimization design for parameters of IHAPF using Jaya algorithm

### Constraints, and objective functions

- Constraint on parameters of *R*_*i*_, *L*_*i*_, *C*_*i*_: values of *R*_*i*_, *L*_*i*_, *C*_*i*_ must be positive and must be ensured to compensate a minimum amount of reactive power and not exceed the maximum limit to be compensated. Therefore:
  $$\begin{array}{c} 0<R_{i}<R_{i-}{}_{\text{max}} \end{array}$$ (1)
  $$\begin{array}{c} 0<L_{i}<L_{i-}{}_{\text{max}} \end{array}$$ (2)
  $$\begin{array}{c} 0<C_{i}<C_{i-\text{max}} \end{array}$$ (3)
- Constraints on DC-link voltage values: a small value of DC bus voltage will not provide enough power to the inverter output, a large value of DC bus voltage will contribute to increased losses during switching. Therefore:
  $$\begin{array}{c} U_{DC-\text{min}}<U_{DC}<U_{DC-\text{max}} \end{array}$$ (4)
- Constraints on reactive power capacity to be compensated: The total compensation capacity *Q*_*b*∑_ must be maximum but not exceeding the maximum capacity to be compensated, which is based on the desired cos*φ* min-max value. Here, the value *cosφ* is usually chosen as the smallest is 0.85, and the largest is 1.0. When *cosφ* = 1, that mean phase shift angle between supply current, and the source voltage is zero. Therefore the total compensation capacity is limited by:
  $$\begin{array}{c} Q_{b}{}_{\sum -\text{min}}<Q_{b}{}_{\sum }<Q_{b}{}_{\sum -\text{max}} \end{array}$$ (5)
- Constraints on PI controller parameters: in this study, we chose PI controller, so the parameters we need to know are *K*_*p*_, and *K*_*i*_. Small *K*_*p*_ parameters will have a slow response but less overshoot, whereas too large *K*_*p*_ will cause instability, and overshoot. The small *K*_*i*_ parameter usually gives a small overshoot at the set, and the large *K*_*i*_ will not minimize the error in steady-state. Therefore, when controlling we often combine two parameters *K*_*p*_ and *K*_*i*_ together, usually, *K*_*p*_ is big, and *K*_*i*_ is small.
  $$\begin{array}{c} 0<K_{p}<K_{pmax} \end{array}$$ (6)
  $$\begin{array}{c} 0<K_{i}<K_{imax} \end{array}$$ (7)
- Objective Functions: The main objective functions considered here are minimum the compensation errors, minimum the phase shift angle between supply current, and source voltage, minimum the total harmonic distortion of supply current.
  $$\begin{array}{c} \left\{ \begin{array}{c} \text{min}THD_{i_{s}}\\ \text{min}error\\ \text{min}(∠\left(u_{s},i_{s}\right)) \end{array}\right. \end{array}$$ (8)

### Flowchart for parameters calculation design for IHAPF using Jaya algorithm

We call *f*(*x*) is the objective function of a minimized problem. n is the dimensional of the problem. There are *popzize* candidate in each iteration are update.

Each iteration, each new solution $X_{i}^{\prime }$ candidate is calculated by formula (9):

$$\begin{array}{c} X_{i}^{{}^{\prime }}=X_{i}+r_{1}(X_{best}-|X_{i}|)\;-r_{2}(X_{worst}-\;|X_{i}|) \end{array}$$ (9)

where, *X*_*i*_ = (*x*_1_, *x*_2_, …, *x*_*n*_) is *i*^*th*^ candidate. *X*_*best*_, and *X*_*worst*_ are the best, and worst candidate respectively. *r*_1_, and *r*_2_ are two random numbers in [0, 1].

**Fig 3:**
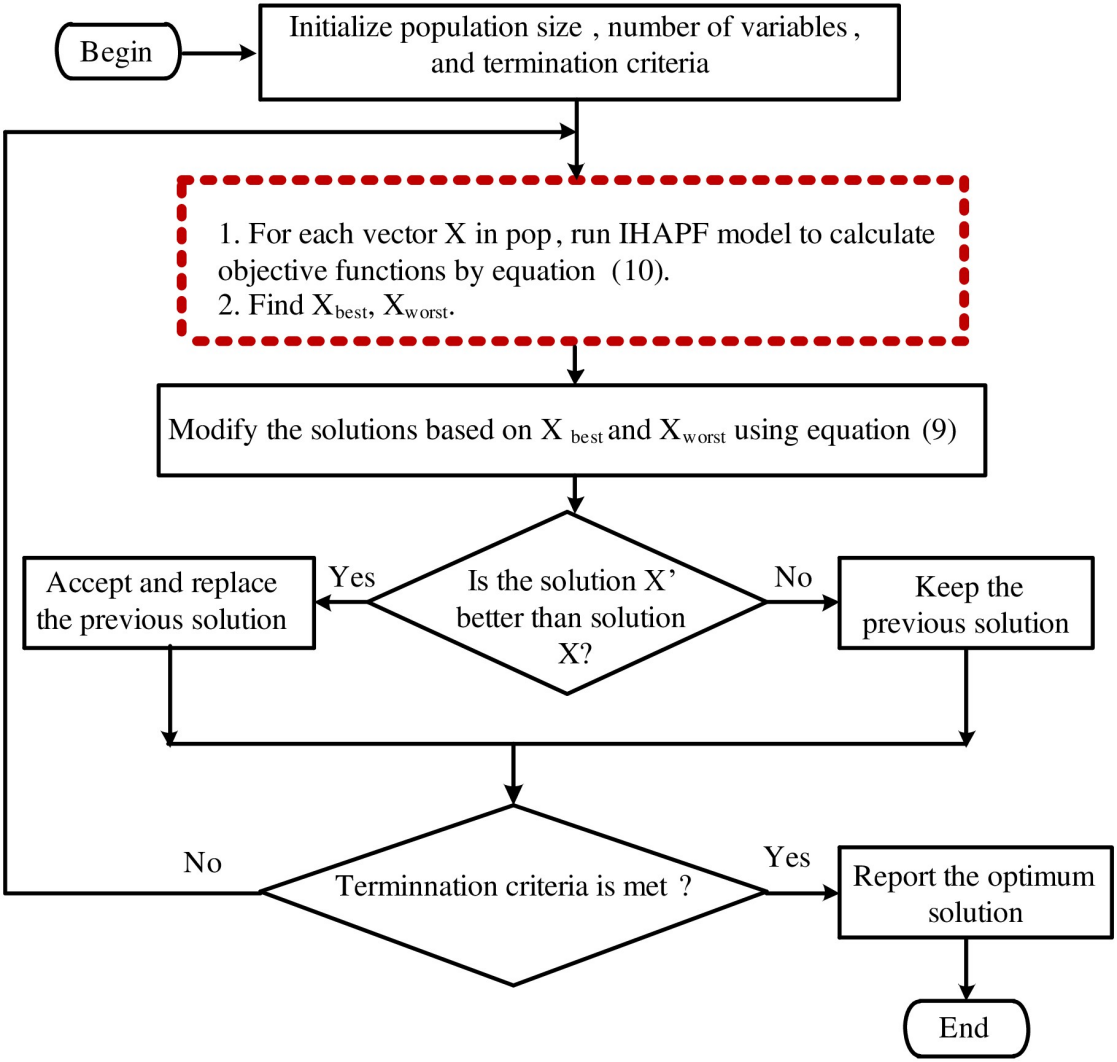
Flowchart for parameters calculation design for IHAPF using Jaya algorithm.

$X_{i}^{\prime }$ is the updated value of *X*_*i*_. The term *r*_1_ (*X*_best_ − |*X*_*i*_|) guide potential solution to move closer to the best solution, and the term *r*_2_ (*X*_worst_ − |*X*_*i*_|) guide potential solution to avoid the worst solution. *X*_*i*_ is replaced by $X_{i}^{\prime }$ if $X_{i}^{\prime }$ gives better function value. Fig 3 shows the flowchart of the proposed algorithm. According two these factors, the algorithm performs the explore, and exploit functions.

The pseudo-code of JAYA algorithm for IHAPF is shown in Algorithm 1.

Algorithm 1: The pseudo-code of Jaya algorithm for IHAPF

1. Initialize population size (*Popsize*), number of variables, and terminated criteria (*Max_Iteration*)

2. **While** Termination criteria is not met **do**

3. For each solution *X*_*i*_ in pop, run IHAPF model;

4. Evaluate fitness functions based on the outputs of IHAPF model and Eq (10);

5. Calculate the *X*_*best*_, and *X*_*worst*_;

6. **For** i = 1 to *popsize* **do**

7. Caculate solution $X_{i}^{\prime }$ by Eq (9);

8. If $X_{i}^{\prime }$ is better, replace *X*_*i*_ by $X_{i}^{\prime }$.

9. **End for**

10. Increase iteration number by one;

11. **End while**.

12. **End Algorithm**

#### Solution presentation

The parameters to search for IHAPF model include: *C*_*F*_ (F), *R*_1_ (Ω), *L*_1_ (H), *C*_1_ (F), *R*_11_ (Ω), *L*_11_ (H), *C*_11_ (F), *R*_13_ (Ω), *L*_13_ (H), *C*_13_ (F), *L*_0_ (H), *U_DC_* (V), *K_p_*, and *K*_*i*_ corresponding to the values *f*_1_, *f*_2_, *f*_3_, *f*_4_, *f*_5_, *f*_6_, *f*_7_, *f*_8_, *f*_9_, *f*_10_, *f*_11_, *f*_12_, *f*_13_, and *f*_14_.

A vector X = (*f*_1_, *f*_2_, *f*_3_, *f*_4_, *f*_5_, *f*_6_, *f*_7_, *f*_8_, *f*_9_, *f*_10_, *f*_11_, *f*_12_, *f*_13_, *f*_14_) is used to presented the solution of the optimization problem.

#### Fitness function

When applied Jaya for IHAPF, the evaluate fitness function is calculated using the output of IHAPF model and Eq 10. For each *X*_*i*_ in pop, the simulation IHAPF model is called. After running the model, *THDi*_*s*_, *error*, and (*u*_*s*_, *i*_*s*_) are produced. In this research, multi-objective problem is converted to single-objective problem. Finally, the fitness function is calculated by Eq 10.

$$\begin{array}{c} Min\;f(X)\;=THDi_{s}+error+∠\left(u_{s},i_{s}\right) \end{array}$$ (10)

## Simulation results, and discussion

To prove the effectiveness of the proposed design flowchart, the parameters needed to search for IHAPF 10kV-50Hz model in Fig 1 will be implemented in turn with four algorithms, the search algorithm using the Jaya, FA, PSO and SA. The parameters to search for IHAPF model include: *C_F_* (F), *R*_1_ (Ω), *L*_1_ (H), *C*_1_ (F), *R*_11_ (Ω), *L*_11_ (H), *C*_11_ (F), *R*_13_ (Ω), *L*_13_ (H), *C*_13_ (F), *L*_0_ (H), *U_DC_* (V), *K*_*p*_, and *K*_*i*_ corresponding to the values *f*_1_, *f*_2_, *f*_3_, *f*_4_, *f*_5_, *f*_6_, *f*_7_, *f*_8_, *f*_9_, *f*_10_, *f*_11_, *f*_12_, *f*_13_, and *f*_14_.

**Fig 4:**
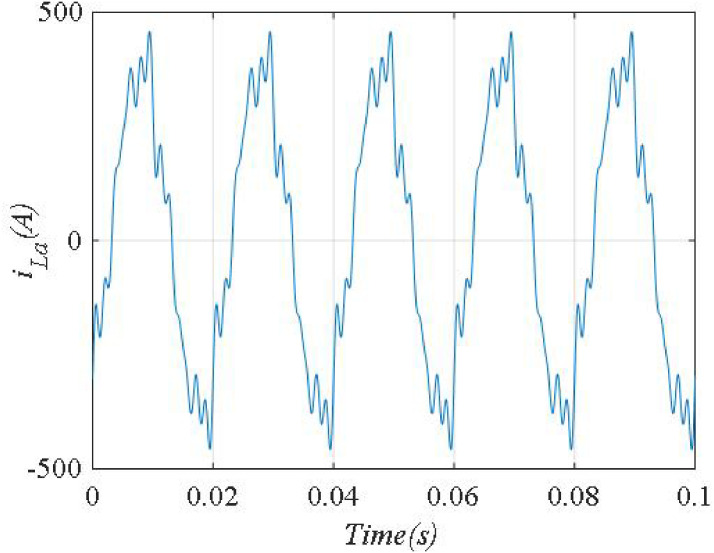
Load current waveform.

**Fig 5:**
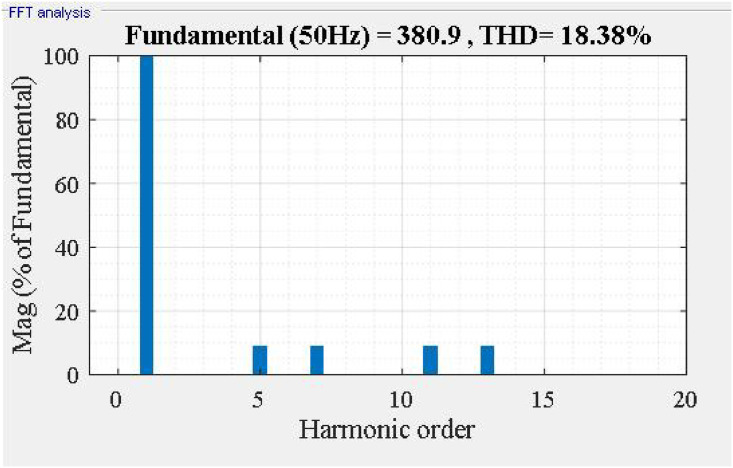
Frequency spectrum of *i_La_*.

Initially, the nonlinear load current, and its frequency spectrum are shown in Figs 4 and 5. The nonlinear load current is lag compared to the source voltage, and the power factor before compensation is 0.64 (lag).

When PSO algorithm is used we have the results as in Tables 1 and 2.

**Table 1:** The results when using PSO algorithm.

	Iteration No. = 1	Iteration No. = 2	Iteration No. = 3	Iteration No. = 4	Iteration No. = 5
*f*_1_	2.924038e-05	2.924038e-05	2.924038e-05	4.914723e-05	4.914723e-05
*f*_2_	0.01048602	0.01048602	0.01048602	0.01712694	0.01712694
*f*_3_	0.01258446	0.01258446	0.01258446	0.01500472	0.01500472
*f*_4_	0.0004634158	0.0004634158	0.0004634158	0.000382653	0.000382653
*f*_5_	0.01810615	0.01810615	0.01810615	0.01059619	0.01059619
*f*_6_	0.001708113	0.001708113	0.001708113	0.001681972	0.001681972
*f*_7_	4.124338e-05	4.124338e-05	4.124338e-05	4.680448e-05	4.680448e-05
*f*_8_	0.01993343	0.01993343	0.01993343	0.01042431	0.01042431
*f*_9_	0.001650619	0.001650619	0.001650619	0.001071445	0.001071445
*f*_10_	1.975492e-05	1.975492e-05	1.975492e-05	3.086599e-05	3.086599e-05
*f*_11_	0.0005561522	0.0005561522	0.0005561522	0.000374114	0.000374114
*f*_12_	785.2274	785.2274	785.2274	745.4446	745.4446
*f*_13_	62.60184	62.60184	62.60184	84.5074	84.5074
*f*_14_	0.7431786	0.7431786	0.7431786	0.7604024	0.7604024
Best Cost	1.9142	1.9142	1.9142	1.5843	1.5843

**Table 2:** The results when using PSO algorithm (continued).

	Iteration No. = 6	Iteration No. = 7	Iteration No. = 8	Iteration No. = 9	Iteration No. = 10
*f*_1_	4.144803e-05	4.377235e-05	4.377235e-05	4.263329e-05	4.263329e-05
*f*_2_	0.01993759	0.01045568	0.01045568	0.01534102	0.01534102
*f*_3_	0.01933229	0.01290587	0.01290587	0.01542507	0.01542507
*f*_4_	0.0006829551	0.0001927401	0.0001927401	0.0003625335	0.0003625335
*f*_5_	0.01360928	0.01561335	0.01561335	0.01937979	0.01937979
*f*_6_	0.001644205	0.001633333	0.001633333	0.001945282	0.001945282
*f*_7_	1.271789e-05	3.496854e-05	3.496854e-05	3.873805e-05	3.873805e-05
*f*_8_	0.01207912	0.01930776	0.01930776	0.01219119	0.01219119
*f*_9_	0.00187596	0.001977769	0.001977769	0.001882403	0.001882403
*f*_10_	2.798992e-05	2.359759e-05	2.359759e-05	2.091486e-05	2.091486e-05
*f*_11_	0.0002712869	0.0003684738	0.0003684738	0.0002357758	0.0002357758
*f*_12_	640.8078	698.5205	698.5205	602.5295	602.5295
*f*_13_	36.87138	64.24956	64.24956	63.69255	63.69255
*f*_14_	0.2350871	0.1970984	0.1970984	0.2389422	0.2389422
Best Cost	1.4197	1.129	1.129	0.91005	0.91005

Elapsed time is 2049.0846 seconds, and the best cost value in steady-state is 0.91005 in iteration No. 10.

**Fig 6:**
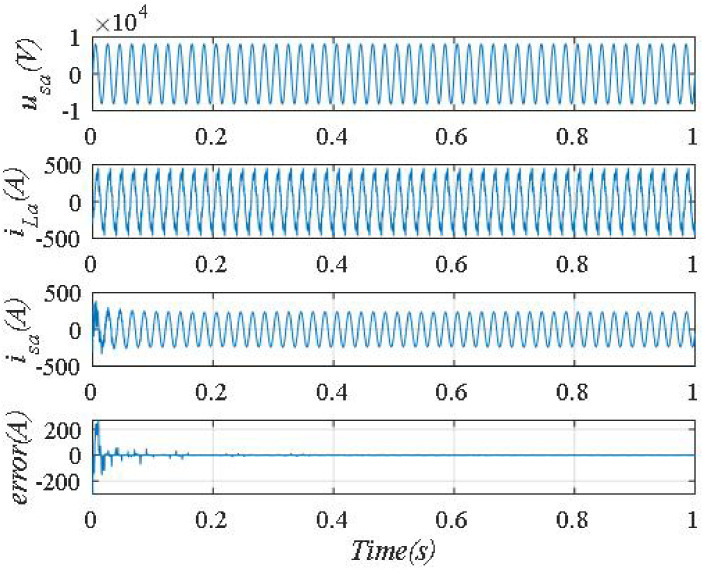
The waveforms corresponding to the best parameter set using the PSO algorithm.

The waveforms corresponding to the best parameter set using the PSO algorithm are shown in Fig 6.

**Fig 7:**
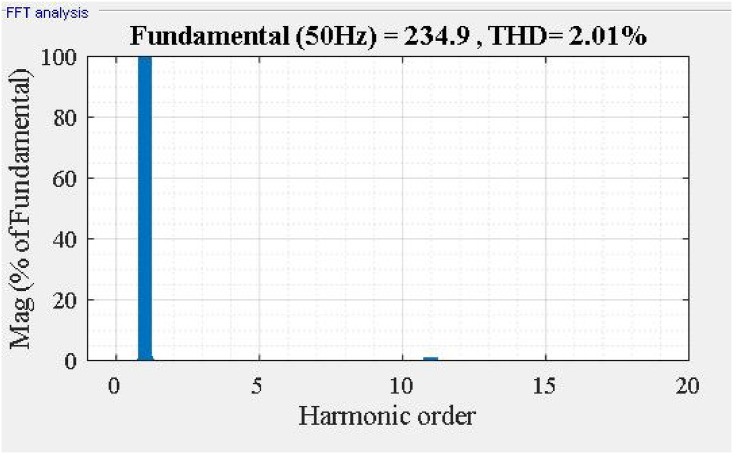
Frequency spectrum of supply current *i_sa_*.

Fast Fourier Transform analysis of the supply current *i*_*sa*_ as shown in Fig 6, we found that $THD_{i_{s}}$ % decreased from 18.38% to 2.01%. The frequency spectrum of supply current *i*_*sa*_ shown in Fig 7.

**Fig 8:**
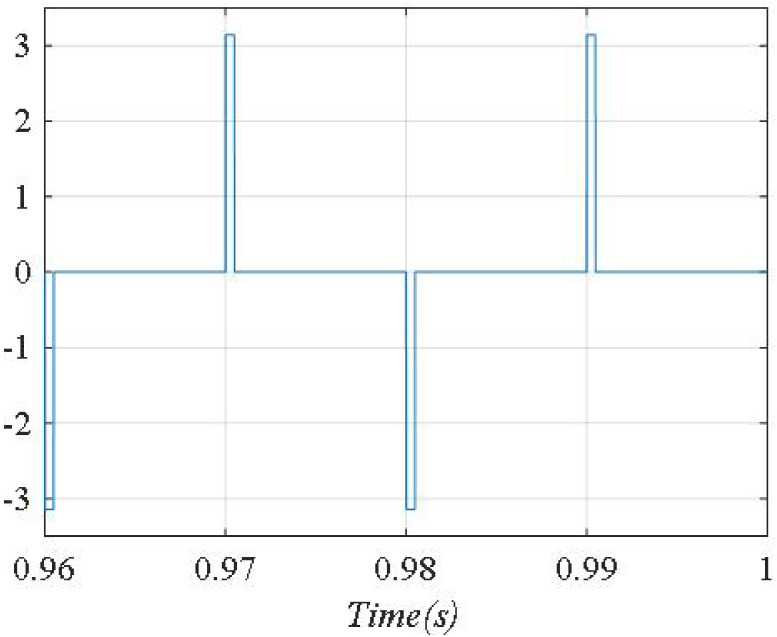
Phase shift angle between *u_sa_* and *i_sa_*.

The phase shift angle between *u*_*sa*_, and *i*_*sa*_ is shown in Fig 8. From Fig 8, we can see that *i*_*sa*_ is lag compared to *u*_*sa*_ by an angle of about 10°, corresponding to the value of the power factor is 0.98(lag). The compensation error at steady state is ±5A.

When the Jaya algorithm is used we have the results as in Tables 3 and 4.

**Table 3:** The achieved results when using the Jaya algorithm.

	Iteration No. = 1	Iteration No. = 2	Iteration No. = 3	Iteration No. = 4	Iteration No. = 5
*f*_1_	5e-5	5e-5	5e-5	5e-5	2.482436e-05
*f*_2_	0.02	0.02	0.02	0.02	0.01185758
*f*_3_	0.01580929	0.01580929	0.01580929	0.01729594	0.0197449
*f*_4_	0.0001	0.0001	0.0001	0.0001	0.0006872732
*f*_5_	0.01046266	0.01046266	0.01046266	0.01092533	0.01519984
*f*_6_	0.001	0.001	0.001	0.001	0.001380426
*f*_7_	3.836756e-05	3.836756e-05	3.836756e-05	5e-5	5e-5
*f*_8_	0.02	0.02	0.02	0.02	0.0181462
*f*_9_	0.002	0.002	0.002	0.002	0.001268174
*f*_10_	4.72922e-05	4.72922e-05	4.72922e-05	5e-5	2.084714e-05
*f*_11_	0.0002	0.0002	0.0002	0.0002	0.0002404516
*f*_12_	500	500	500	500	630.5891
*f*_13_	39.11946	39.11946	39.11946	44.83766	60.01977
*f*_14_	0.3905476	0.3905476	0.3905476	0.1	0.1
Best Cost	2.1933	2.1933	2.1933	2.0895	1.7277

**Table 4:** The achieved results when using the Jaya algorithm (continued).

	Iteration No. = 6	Iteration No. = 7	Iteration No. = 8	Iteration No. = 9	Iteration No. = 10
*f*_1_	3.238689e-05	3.285666e-05	3.285666e-05	4.116398e-05	4.116398e-05
*f*_2_	0.01	0.01	0.01	0.01367639	0.01367639
*f*_3_	0.02	0.02	0.02	0.02	0.02
*f*_4_	0.0007	0.0007	0.0007	0.0001	0.0001
*f*_5_	0.01796313	0.02	0.02	0.01709288	0.01709288
*f*_6_	0.002	0.002	0.002	0.002	0.002
*f*_7_	5e-5	5e-5	5e-5	4.280462e-05	4.280462e-05
*f*_8_	0.01	0.01	0.01	0.01	0.01
*f*_9_	0.002	0.002	0.002	0.001761725	0.001761725
*f*_10_	2.552435e-05	1e-5	1e-5	2.73808e-05	2.73808e-05
*f*_11_	0.0002264428	0.0002528856	0.0002528856	0.0003568722	0.0003568722
*f*_12_	800	800	800	659.9763	659.9763
*f*_13_	56.80504	100	100	100	100
*f*_14_	1	1	1	0.6063943	0.6063943
Best Cost	1.1001	0.93592	0.93592	0.73731	0.73731

Elapsed time is 1764.328972 seconds, and the best cost is 0.73731 in iteration No. 10.

**Fig 9:**
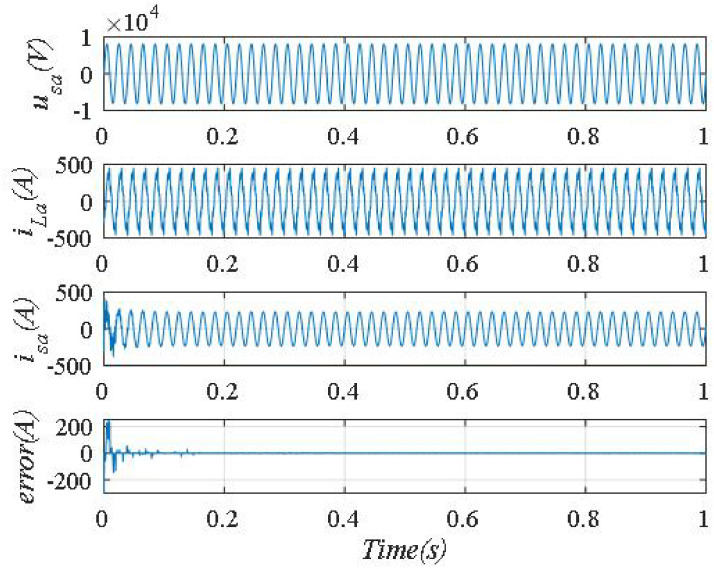
The waveforms corresponding to the best parameter set using the Jaya algorithm.

The waveforms corresponding to the best parameter set when using the Jaya algorithm are shown in Fig 9.

To do a comparison, the experiment is run and demonstrated in Table 5. The parameters for the algorithms in Table 5 are setting as following: In three algorithms, population size is 3, and Iteration is 25. For PSO, *c*_1_ equal to 1.2, *c*_2_ equal to 1.2. For simulated annealing, all other parameters are set as [38]. For FA, *α* = 1.0, *β*_0_ = 1.0, *γ* = 0.01, *θ* = 0.97. Each algorithm is run 15 times. In Table 5, Mean, best, worst, and stdEV are average, best, worst, and standard deviation of 15 runs. The results conducted by the Jaya algorithm are better than the others.

**Table 5:** The comparison results of FA, PSO, SA and Jaya algorithms.

	Best	Worst	Mean	stdEV
Jaya	0.0023	0.0162	0.0042	0.003
FA	0.0067	0.0823	0.0238	0.023
PSO	0.0032	0.0060	0.0038	0.001
SA	0.004	0.1095	0.0565	0.040

**Fig 10:**
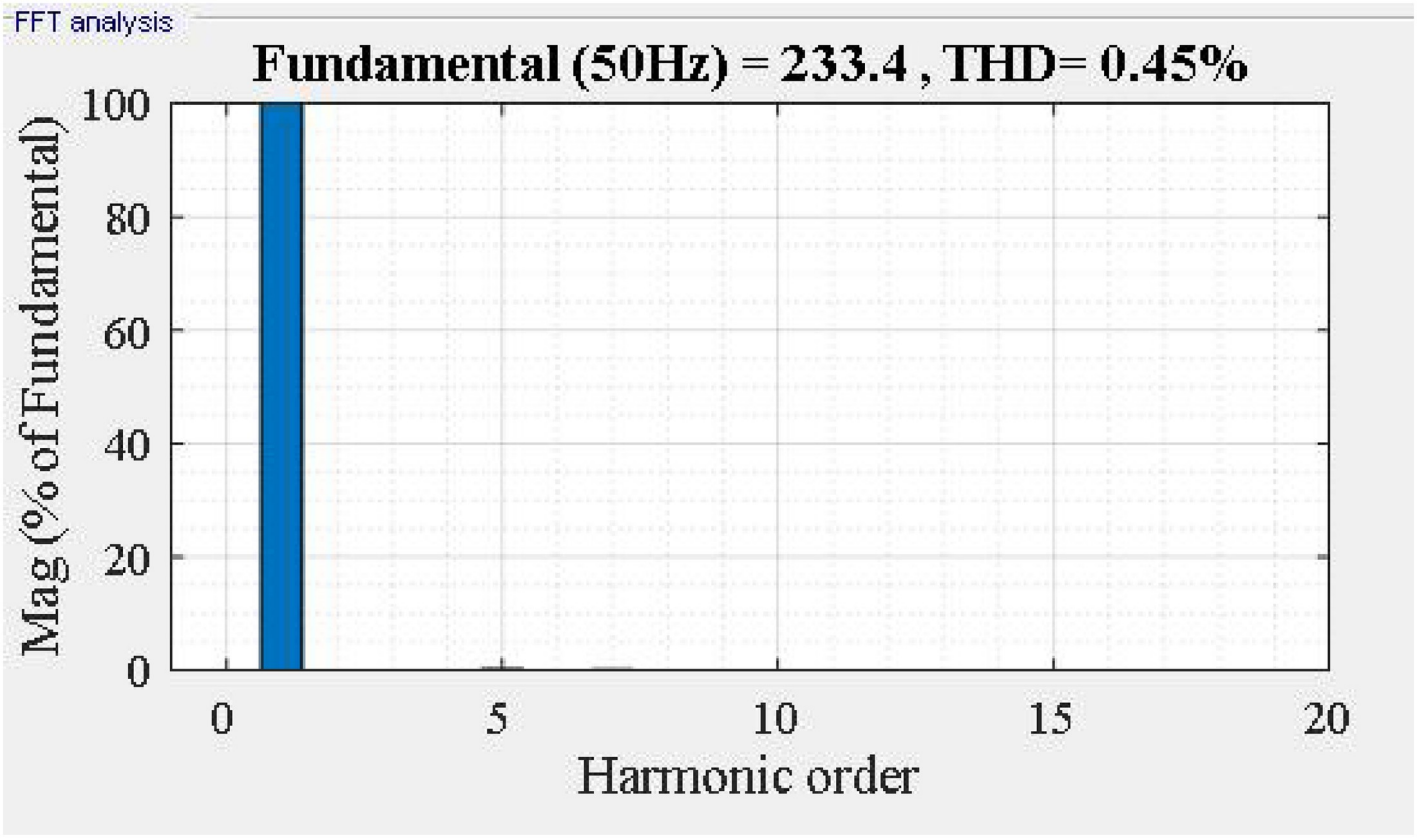
Frequency spectrum of supply current isa with Jaya algorithm.

Fast Fourier Transform analysis of the supply current *i*_*sa*_ in Fig 9, we can see that the total harmonic distortion of *i*_*s*_% decreased from 18.38% to 0.45%. The frequency spectrum of supply current *i*_*sa*_ shown in Fig 10.

**Fig 11:**
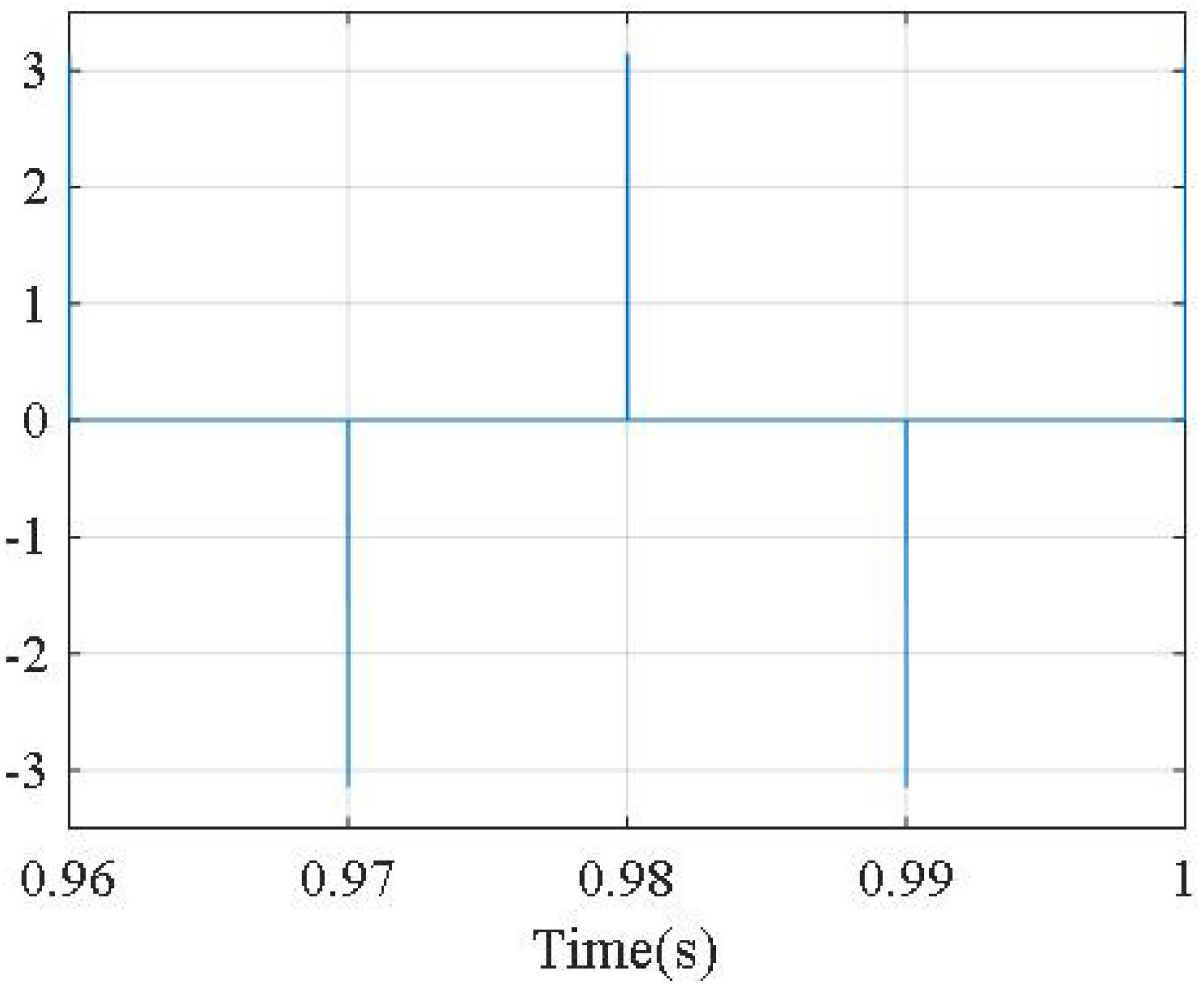
Phase shift angle between *u*_*sa*_, and *i*_*sa*_.

The phase shift angle between *u*_*sa*_, and *i*_*sa*_ is shown in Fig 11. From Fig 11, we can see that phase shift angle between *u*_*sa*_, and *i*_*sa*_ is almost zero, corresponding to the value of the power factor cos*φ* = 1. The compensation error is ±3A.

### Wilcoxon rank-sum test

With the observable measure, you’ll be able to prove beyond a shadow of a doubt that the results aren’t random. The non-parametric Wilcoxon statistical test is applied, and the resulting p-values are also presented as significance metrics. When comparing Jaya to FA, PSO, and SA, any p-values less than 0.05 indicate statistically significant superiority of the outcomes. p values are presented in Table 6.

**Table 6:** p values of the Wilcoxon rank-sum test over 15 runs.

FA	PSO	SA
2.30E-05	2.74E-02	2.30E-05

In summary, from the above analysis results, we realize that the Jaya search algorithm is more effective than firefly algorithm, PSO search algorithm, and the simulated annealing algorithm in minimizing the total harmonic distortion of supply current, minimum phase shift angle between source voltage, and supply current, and minimum compensation error in steady-state. Moreover, Jaya algorithm also has computing time faster than PSO algorithm.

## Conclusion

The paper introduced a new multi-objective optimization design flowchart using the Jaya algorithm for IHAPF. The highlight of the proposed algorithm flowchart is that it can determine all the parameters of the IHAPF system with a short search time, and very good results. Compared with the Firefly Algorithm, PSO algorithm, and the Simulated Annealing algorithm, the simulation results have proved that: Jaya algorithm is better at minimizing compensation errors, minimum phase shift angle between source voltage, and supply current, minimum total harmonic distortion of supply current, and less search time.
